# The development and implementation of a guideline-based clinical decision support system to improve empirical antibiotic prescribing

**DOI:** 10.1186/s12911-022-01860-3

**Published:** 2022-05-10

**Authors:** H. Akhloufi, H. van der Sijs, D. C. Melles, C. P. van der Hoeven, M. Vogel, J. W. Mouton, A. Verbon

**Affiliations:** 1 Department of Medical Microbiology and Infectious Diseases, Erasmus MC University Medical Center, PO Box 2040, 3000 CA Rotterdam, The Netherlands; 2 Department of Internal Medicine, Division of Infectious Diseases, Erasmus MC University Medical Center, Rotterdam, The Netherlands; 3 Department of Hospital Pharmacy, Erasmus MC University Medical Center, Rotterdam, The Netherlands

**Keywords:** Clinical decision support system, Reporting framework, Antibacterial, Antibiotic stewardship

## Abstract

**Background:**

To describe and evaluate a clinical decision support system (CDSS) for empirical antibiotic therapy using a systematic framework.

**Methods:**

A reporting framework for behavior change intervention implementation was used, which includes several domains: development, evaluation and implementation. Within the development domain a description is given of the engagement of stakeholders, a rationale for how the CDSS may influence antibiotic prescribing and a detailed outline of how the system was developed. Within the evaluation domain a technical validation is performed and the interaction between potential users and the CDSS is analyzed. Within the domain of implementation a description is given on how the CDSS was tested in the real world and the strategies that were used for implementation and adoption of the CDSS.

**Results:**

Development: a CDSS was developed, with the involvement of stakeholders, to assist empirical antibiotic prescribing by physicians. Evaluation: Technical problems were determined during the validation process and corrected in a new CDSS version. A usability study was performed to assess problems in the system-user interaction. Implementation: In 114 patients the antibiotic advice that was generated by the CDSS was followed. For 54 patients the recommendations were not adhered to.

**Conclusions:**

This study describes the development and validation of a CDSS for empirical antibiotic therapy and shows the usefulness of the systematic framework for reporting CDSS interventions. In addition it shows that CDSS recommendations are not always adhered to which is associated with incorrect use of the system.

**Supplementary Information:**

The online version contains supplementary material available at 10.1186/s12911-022-01860-3.

## Background

To improve quality of antibiotic prescriptions and thereby help to control the emergence and spread of antibiotic resistance, several Antibiotic Stewardship Programs (ASPs) have been developed [1–3]. These programs are ideally administered by an Antimicrobial Stewardship Team (AST), a multidisciplinary team composed of an infectious disease physician, a clinical pharmacist with infectious diseases training, a clinical microbiologist, an infection control professional and a hospital epidemiologist [4]. One of the most important objectives of these ASPs is the use of empirical antibiotic therapy according to guidelines [5, 6], which has been associated with a relative risk reduction for mortality [5]. However, whereas empirical antibiotic therapy has been shown to be significantly more appropriate after consultation of an infectious disease specialist [10], for the majority of patients antibiotics are prescribed by their attending physician.

Using specific strategies to promote antibiotic prescribing according to the guidelines seems necessary, since it is generally not effective to passively disseminate guidelines [7]. Clinical decision support systems (CDSSs) can link patient data with an electronic knowledge base with clinical guidelines to improve decision making. As the use of electronic medical records increases and new information technologies are being developed, CDSSs for antimicrobial stewardship have gained widespread interest [8–10]. As part of an ASP, CDSSs can play an important role by taking over part of the activities of an AST. This is attractive given the fact that ASTs are labor intensive and thus expensive [11, 12].

Several CDSS to improve empirical antibiotic prescribing in hospitalized patients have been developed and assessed over the years [13–18]. These systems have the potential to improve empirical antibiotic prescribing [13–17], but the development of these systems has been poorly reported. The need for detailed description of system design has been addressed [9]. A low level of CDSS use is found in several studies [8, 19–23]. Unfamiliarity with the system and a vague description or no description at all of the development of these systems may play a role in the lack of success of CDSS in clinical practice until now. In addition the literature describes a need for a systematic reporting framework, because of a heterogeneous and disjointed approach to reporting CDSS interventions [8, 24, 25]. In this study we describe in detail the development, evaluation and implementation of a CDSS following the reporting framework developed by Rawson et al., which is adapted from the Stage Model of Behaviour Intervention Development [8]. In this model 5 stages of intervention research are highlighted [26]. Stage 0 is about understanding the basic principles of behavior change for the intervention.

Stage I includes all activities related to the creation of a new intervention, or the modification, adaptation, or refinement of an existing intervention (Stage IA), as well as feasibility and pilot testing (Stage IB).

Stage II research consists of efficacy testing of promising behavioral interventions in research settings, with research therapists/providers. Stage III is similar to Stage II research, except that instead of research providers and settings, it consists of testing in a community context while maintaining a high level of control necessary to establish internal validity. Stage IV is effectiveness research. Stage IV research examines behavioral interventions in community settings, with community therapists/providers, while maximizing external validity.

Stage V is implementation and dissemination research.

Using this framework a CDSS intervention can be evaluated in a systematic manner taking into account several domains, including development, evaluation and implementation. This study describes the development and validation of a CDSS for empirical antibiotic therapy. It is, to our knowledge, the first to use this framework to report on a CDSS intervention for antimicrobial therapy and evaluate the usefulness of it.

## Methods

### Setting

This study was conducted at the Erasmus MC, University Medical Centre, a 1125 bed tertiary care center in Rotterdam. A total of 31.923 patients were admitted to this hospital in 2018. The Erasmus MC uses an electronic health record (EHR) with integrated computerized prescriber order entry (CPOE). The Department of Medical Microbiology and Infectious Diseases of this hospital provides an active Infectious Diseases (ID) consultation service, in which ID consultants pro-actively give the attending physicians recommendations about antibiotic use.

### Clinical decision support system—development

A web-based clinical decision support system for empirical antibiotic therapy for adult hospitalized patients was developed by a multidisciplinary team. This team consisted of an ID specialist, clinical microbiologists, a hospital pharmacist experienced in decision support, an Information Technology (IT) team and a researcher.

The CDSS was iteratively developed through biweekly meetings between the multidisciplinary team. During these meetings several items were discussed. Items that were discussed included which and how extra information should be provided in the system to increase the ease of use and limit errors, such as the CURB-65 score for pneumonia severity. This was done in light of the many residents and fellows working in our hospital. Other important items that were discussed were which known cultures should be presented in the system (all cultures or only recent ones, all cultures or only those relevant for the working diagnosis) and how recent the automatically extracted data should be (eGFR value and neutrophil value). Other discussed items were for example the formulation of questions, how we could show the user the progression of her or his advice request, which information should accompany the generated antibiotic advice and from what age the existence of pregnancy should no longer be asked for. Consensus was needed for optional and extra manual input by the physicians when using the CDSS, since information in the hospital information system can be missing or inaccurate. For example fluctuating information, such as the weight or renal function, may not be continuously updated and therefore be outdated at the time of CDSS use.

During the development of the system, different infectious disease consultants were asked for feedback resulting in improvements in lay-out or functionality. The developed CDSS is based on the local antibiotic treatment guidelines, which are in line with the national guidelines (https://adult.swabid.nl). By generating patient specific antibiotic advices, based on relevant guidelines, this system makes it physicians easy to appropriately prescribe antibiotics. The system takes into account all relevant parameters, such as kidney function, culture history and pregnancy. This decreases the risk of overlooking a relevant parameter and increases the chance of optimal antibiotic prescribing. The CDSS was based on frequently occurring infections in our hospital. In addition we also included several infectious diseases on request of physicians. We performed a usability study as part of the development and evaluation phase [27], resulting in stakeholders being engaged in the further development and fine-tuning of the CDSS. The results of the usability study enabled us to make the CDSS more specific to users’ needs (for example by adding calculators). A description of the usability study is given under the heading ‘Clinical decision support system—evaluation’.

### Clinical decision support system—evaluation

We used two steps to evaluate the CDSS before we implemented the system in clinical practice. During the first step we used a retrospective technical validation to confirm that the used CDSS parameters were correctly linked to the data in the EHR. During the second step a usability study was performed using realistic clinical scenarios to assess the interaction between the potential end user and the developed system.

#### Step 1

Flowcharts were first developed on paper and checked for correctness by the development team. Thereafter, the CDSS was built and technically and clinically tested using real patient data to trigger an antibiotic advice. Automatically extracted data were checked on correctness in our EHR. All generated antibiotic recommendations were manually and automatically checked on correctness using local current guidelines and developed flowcharts.

#### Step 2

To improve lay-out and functionality we performed a usability study [27]. We used a user-based usability evaluation method, where participants had to verbalize their thoughts during the execution of a set of specified tasks using the CDSS. Sessions were recorded and analyzed afterwards by 3 evaluators using an augmented classification scheme. The severity of the identified usability problems were rated and the potential impact of these problems on the final task outcomes was assessed.

After these tests the CDSS was made available for use, by providing the link to the web-based system in our hospital information system. An overview of the CDSS characteristics, development and evaluation can be found in Table 1.

**Table 1:** Description and evaluation of the clinical decision support system for empirical antibiotic therapy (following the identified reporting criteria by Rawson et al. [8])

*Description of decision support tool*	
Type of decision support provided	Antibiotic (empirical) prescribing Dose optimization Duration of therapy Route of administration
Platform on which it is provided	Web-based
Infrastructure	Rule-based
*System development*	
Rationale for development	Makes it easy to do right by generating patient specific antibiotic advices, based on relevant guidelines Decreasing the risk of overlooking a relevant parameter in antibiotic prescribing Stakeholders were involved in the development of the system with the use of a usability study. Diagnoses were included in the system on request of stakeholders
Previous feasibility/pilot testing	A usability study was performed to assess the interaction between the system and user. With this study we also assessed whether the generated advices would be followed and identified potential negative outcomes/errors
Evidence supporting evaluation	A usability study provides detailed insight into usability problems experienced by end-users of the system. It also provides insight in the causes of identified problems
How the tool is implemented	A demonstration was given before implementation The use of the CDSS was regularly promoted by visiting departments. Medical pocket cards were developed as promotional material for the system An active infectious disease consultancy service system is provided in the hospital where the CDSS is implemented. ID consultants were instructed to remind physicians to use the CDSS
*Study design*	
Justification for study design	Descriptive/observational study. This study design is selected to describe the use of the developed CDSS and adoption of its recommendations
Outcome measure selection	Evaluation of the adoption of generated advices

### Clinical decision support system—implementation

The CDSS was demonstrated on the different clinical departments before implementation. During the implementation period (December 2016 until May 2017) the departments were visited on a regular basis to promote the use of the CDSS and answer questions regarding the system. In addition we designed medical pocket cards as promotional material for the system. During the implementation period, each advice that was generated by the CDSS was checked the same day by a clinical infectious disease consultant.

### Data collection

To assess the performance of the CDSS during the implementation period, a data file was created with relevant patient data, which was automatically retrieved from our hospital information system. All adult patients in all clinical departments of the Erasmus MC, with the exception of 1-day admissions, using at least one antibacterial drug for systemic use (ATC code starting with J01) in the implementation period of the CDSS were selected. Patients that received only prophylactic antibiotics were excluded. The following antibiotic drugs were defined as prophylaxis: all antibiotics given for a duration of less than 48 h, cotrimoxazole at a dose of 480 mg and cefazolin started pre-, intra-, or postoperatively without another clear indication (manually assessed). Antibiotics given regarding a prophylactic protocol, such as selective decontamination of the digestive tract, antibiotics for patients with neutropenia, chronic obstructive pulmonary disease (COPD), and pheneticillin within a period of 2 years after splenectomy were also defined as prophylaxis. Relevant data such as age, sex, ward, prescribed antibiotic(s), infectious disease consultations and advised antibiotic(s) by the CDSS were automatically retrieved. For every patient for whom the CDSS was used, it was assessed by chart review whether the antibiotic(s) advised by the CDSS were (partly) followed or not. If only one of the recommended drugs or a different route or dosage regimen was prescribed, this was categorized as partly followed. Cases of doubt were discussed by two of the researchers (HA and AV). Since not all antibiotic guidelines were present in the CDSS, we assessed in how many patients the system could be used. In our data file with all 3349 patients that received at least one antibiotic drug for systemic use during the implementation period, we randomly selected 248 patients to manually check whether the patients had a diagnosis for which the CDSS could have been used. In this proportional stratified random sample of 248 patients all departments were reflected.

The study was carried out in accordance with relevant guidelines and regulations and according to the Dutch Medical Research in Humans Act, medical ethical approval was not required and patients did not need to provide informed consent, since their data were handled anonymously by the researcher.

## Results

### Clinical decision support system—development

**Fig. 1:**
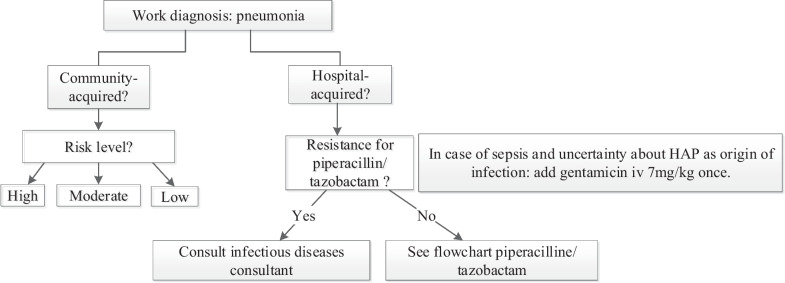
Part of the flowchart developed for the working diagnosis pneumonia. For the complete flowchart of high and moderate risk community acquired pneumonia see Additional file 1: Figure S1. HAP is hospital acquired pneumonia. Risk level was assessed using the CURB-65 score

**Fig. 2:**
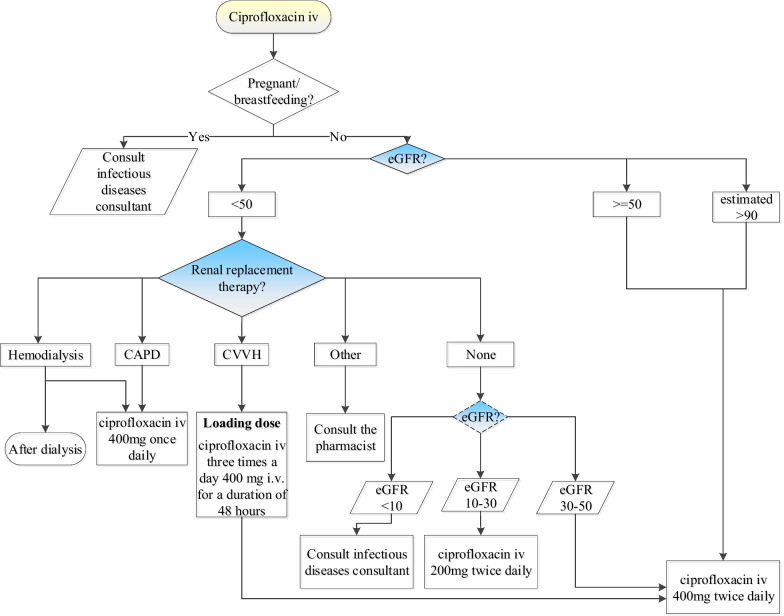
The flowchart for ciprofloxacin iv with all relevant information that the CDSS takes into account

Our CDSS included the following diagnoses: sepsis, pneumonia, urinary tract infections, fever of unknown origin (with suspicion of bacterial infection), meningitis, secondary peritonitis and liver abscess. The diagnoses secondary peritonitis and liver abscess were included on request of physicians. For each diagnosis, a flowchart to map relevant information for the choice and duration of the antibiotic was developed such as the working diagnosis and, for example, whether a pneumonia was community or hospital acquired (Fig. 1). To determine the right dose and dosing interval, flowcharts were designed for different antibiotics by mapping relevant factors such as renal function, weight or body mass index and pregnancy (Fig. 2). In addition, factors such as allergies, and antibiotic susceptibility in the previous 6 months were incorporated in the CDSS in order to deviate from the first choice empiric antibiotic if necessary. The clinical decision support system was built as an interactive system, which had to be activated by the physician. It extracted automatically as much relevant patient information as possible from our hospital information system to which it was connected. Automatically extracted patient data were patient identification number, birth date, sex, admission ward, culture history, kidney function and absolute neutrophil count. To generate an appropriate antibiotic advice some information input, which could not be automatically extracted from the hospital information system, was needed from the prescriber such as the working diagnosis.

### Clinical decision support system—evaluation

Step 1. The recommendations by the CDSS were all in accordance with the local antibiotic guidelines. Some bugs in the CDSS, such as an incorrect threshold value and no generated advice in the end screen, were found with the automatic technical tests, which were corrected in a new version of the CDSS.

**Fig. 3:**
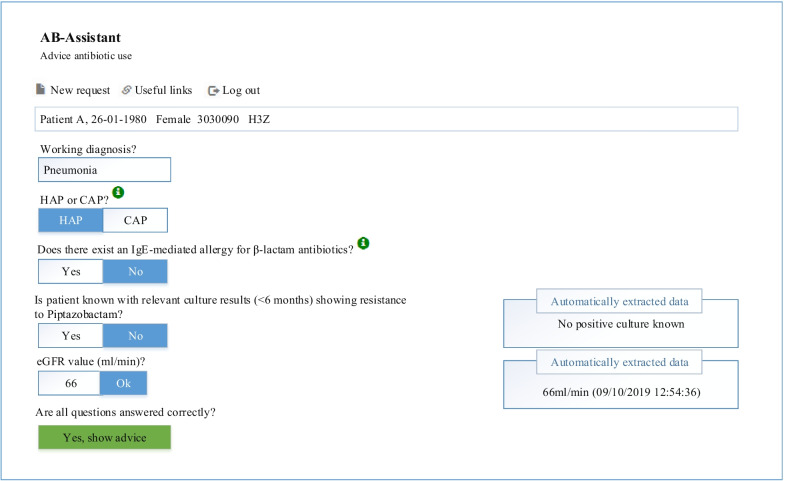
The clinical decision support system for empirical antibiotic therapy for pneumonia. HAP is hospital acquired pneumonia. CAP is community acquired pneumonia. Patient data are not from an existing patient

Lay-out. For all diagnoses, the physician has to manually fill in the working diagnosis and answer a few questions in the different predefined pathways (Fig. 3). We added mouse over information for items that might not be clear, such as what an IgE mediated allergy is or needed knowledge of criteria, for example severity of CAP. In the example shown in Fig. 3, after answering the question about allergy the choice of antibiotic is clear and should be refined by incorporating culture results. The appropriate antibiotic is given and the physician is obligated to check the antibiogram of previous cultures before he/she can proceed with the program (for an example see Additional file 1: Figure S2). Only culture results from the previous 6 months were presented. Antibiotics were preselected by the CDSS based on the working diagnosis and relevant parameters. Dosing regimen was refined by using the eGFR and in case of gentamicin using (ideal) body weight. Only eGFR values were presented with date and time of eGFR determination if determined less than 1 week before consulting the CDSS.

Step 2. During the usability study a total 51 usability problems were identified, grouped into 29 different categories. Most (n = 17/29) of the problems were cosmetic problems or minor problems. Eighteen (out of 29) of the usability categories could have an ordering error as a result [27]. To improve this the system was redesigned by enlarging information icons, introducing calculators, retrieving more patient information automatically from the hospital information system and introducing new options, such as the option to review the culture history in the final screen when an antibiotic advice is generated.

### Clinical decision support system—implementation

During the implementation period of the CDSS, 3349 patients received at least one antibiotic for systemic use. In the stratified random sample of 248 admitted patients 100 patients received at least one antibacterial drug as empirical antibiotic therapy for one of the diagnosis that are included in the CDSS, which is equivalent to 40.3% (CI_95%_ [34.3,46.3]) of patients. That means that of the 3349 patients that received at least one antibiotic for systemic use, 1349 patients received this antibiotic(s) as empirical antibiotic therapy for one of the included diagnosis in the CDSS. The CDSS was used 184 times, of which 15 times for patients who did not have any signs of infection or were not admitted to the hospital (trying out/testing the system). Thus the system was used for 12.5% (184-15)/1349) of patients for which it could be used. The median age of patients for which the CDSS was used was 64 years and 44.4% (75/169) was female. The CDSS was mostly consulted for the diagnosis pneumonia (hospital acquired and community acquired) (62/169), followed by urinary tract infection (58/169). The CDSS was mainly used by physicians working at the internal medicine department. All recommendations given by the CDSS were correct for the presumed working diagnosis.

### Clinical decision support system—recommendations and adoption

**Fig. 4:**
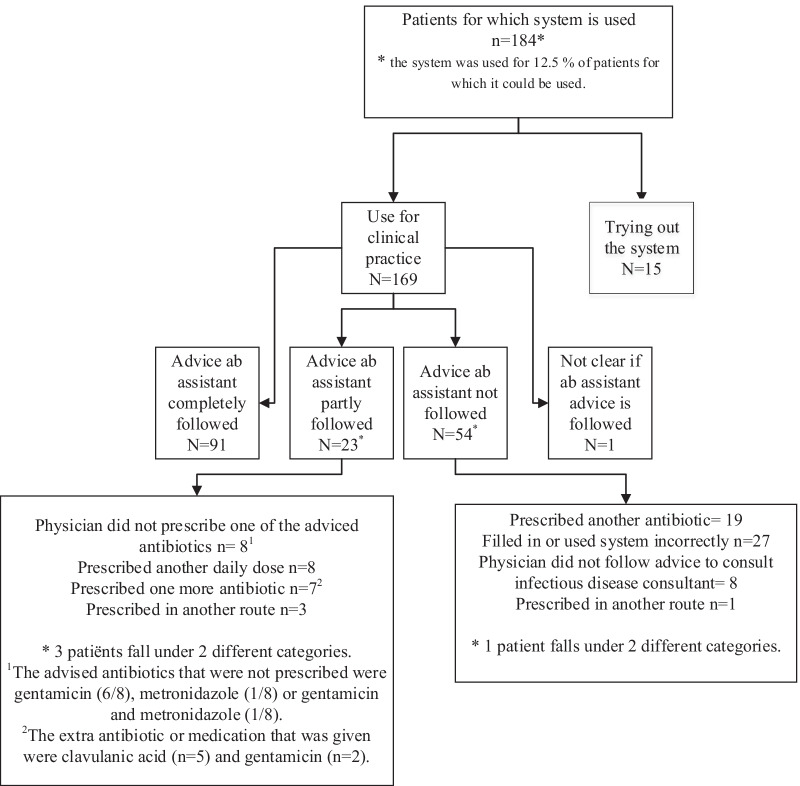
Use of the CDSS and adoption of its recommendations

The CDSS was used to generate antibiotic advice for clinical practice for 169 patients: for 141 patients an antibiotic advice was given, including dose and route and for 28 patients the advice was to consult an infectious disease consultant. The most commonly recommended drug for pneumonia was piperacillin with tazobactam and for urinary tract infections nitrofurantoin. In 114 patients (67,4%) the advice that was generated by the CDSS was completely (n = 91) or partly (n = 23) followed. We found several explanations for the deviation from the advised antibiotics(s) by the CDSS (Fig. 4). Some physicians filled in or used the system incorrectly, they for example tried to fit in a diagnosis or a wrong diagnosis was filled in. We also found that a reconsideration of the working diagnosis/differential diagnosis or the wish of the physician to prescribe an oral alternative instead of the advised intravenous antibiotic could explain the discrepancy in prescribed antibiotic(s) and the generated advice by the CDSS. The same applies to not correctly filled in allergies (not manually entered or entered while no allergy existed), the use of the system for directed therapy instead of empirical therapy and the use of the system while a bacterial infection was absent. The 15 patients that were used to test/try out the CDSS did not have any signs of infection or were not admitted to the hospital.

## Discussion

We have developed, validated and implemented a CDSS to assist and improve empirical antibiotic choices in adult hospitalized patients. In line with the proposed systematic framework by Rawson et al. [8], this study describes the development and validation of a CDSS for empirical antibiotic therapy and shows the usefulness of a systematic framework for reporting CDSS interventions. The CDSS was mostly consulted for the diagnosis pneumonia, and urinary tract infection. The advice of the CDSS was 100% correct given the data that were filled in. In 67.4% the advice that was generated by the CDSS was followed (completely or partly). For cases in which the CDSS advice was not followed by the physician, half of them filled in or used the system incorrectly. A CDSS for empirical antibiotic therapy has the potential to increase guideline adherent therapy. However, CDSS recommendations are not always adhered to and this could be explained mostly by incorrect use of the system.

This study is, to our knowledge, the first study that uses systematic approach, in which the development, validation and implementation of a CDSS for empirical antibiotic therapy are described in detail. The used reporting framework provides a structured overview of many important aspects of a CDSS intervention. It gives an understanding of the rationale for why and how a CDSS was developed and how its effectiveness was evaluated [26, 28]. Following this framework ensures reporting on these different aspects and when applied also by others will enable a more easy comparison between the different CDSSs. Several important aspects are not included in this framework, such as: the composition of the team that developed the CDSS, type of CDSS (active or a passive), guidelines on which the CDSS is based, rationale for using these guidelines, commercial or noncommercial CDSS, setting for which the CDSS was developed. We propose that these key components should also be considered when reporting on CDSS interventions.

In the implementation domain we found that a factor that compromised the potential of the CDSS is that not all recommendations were followed. Because in half of these non-followed recommendations the system was filled in or used incorrectly, training in its use is recommended. During this training attention can be given to assessing the relevance of previous cultures, because this can be difficult. Physicians could have been testing/trying out the system using data of the admitted patients with signs of infection. For this reason it is recommended to provide test patients, specially created for this purpose. Incorrect use of the system was because physicians for example tried to fit in a diagnosis. In the development phase we decided to include the most common infections in our CDSS. Such an approach has also been applied in another study in which a need for assistance with empirical antibiotic choices when less common infections were present was expressed [14]. In this study the following foci of infection were included: blood, wound, lower respiratory tract, abscess and urinary tract. In our and their study the most common diagnosis for which the CDSS was used was respiratory tract infection. The inclusion of more diagnoses is recommended to tackle the problem of incorrect use of the system because physicians try to fit in a diagnosis. However, the choice of diagnoses accompanied by an antibiotic advice should be in balance with the amount of work associated with the inclusion of the specific infection and use of the system for these extra infections.

A similar non-adherence to CDSS recommendations has been described before [20], [29]. However a wide range in adherence to CDSS recommendations has been reported [22], which may be explained by differences in CDSS usability, type of CDSS recommendation (fine-tuning or a complete antibiotic advice), local hospital environment and culture [30]. CDSS may be more effective when the advice is provided automatically, more patient specific and when it is combined with other interventions [31]. Other explanations for not following CDSS recommendations are the complexity of patient cases, other infectious disease diagnoses that present similarly or a reconsideration of the working diagnosis [18]. In our study, the wish of physicians to prescribe an oral alternative and/or reconsideration of the working diagnosis were also reasons for not following the CDSS recommendation. Monitoring reasons to deviate from CDSS recommendations is important to further optimize (the implementation of) a CDSS.

Despite the development by a multidisciplinary team with involvement of the end-user using a usability study, subsequent improvement of the system, the system being easy to use and promotion of use in clinical practice, the system was used in only 12.5% of patients for which it could have been used. For this low use different explanations exist, such as users not being convinced of the validity (correctness and completeness) of the information provided by the system and the fear of compromising their professional autonomy. Also the attitude of physicians towards existing guidelines/quality of evidence on which the CDSS is based may play a role, which in our case differed for several aspects of the recommendations [30, 32]. To tackle these barriers we have included all relevant sources of information in the CDSS, included the option to override automatically generated parameters on which the final antibiotic advice would be based and asked for manual input of data by the physician. Another explanation for the low use of the CDSS, which is more likely, is that it is a passive and new system. At the moment of empirical antibiotic prescribing, physicians had to realize that a CDSS could assist them. A reminder in the CPOE and direct access from the CPOE (fitting in the work process) could be an option to improve CDSS use. The change of antibiotic prescribing habits and culture could also have been a barrier to the use of the CDSS. Related to this is the availability of an active ID consultation service system in our hospital, which the attending physicians are used to consult. The 6-month time period may also have been too short to improve use/uptake of the CDSS, since it seems that continued use of these systems improves their acceptance [18, 29, 33–35]. To gain insight in the specific barriers to CDSS use in our hospital further research is useful.

A strength of this study is that the CDSS was operated by the physicians themselves, which gives insight in their use of this CDSS after implementation and in the acceptance of the recommendations. In many studies regarding CDSS for empirical antibiotic prescription, the system was not used by the end-users, the attending physicians, who are the most frequent antibiotic drug prescribers [13, 15, 16, 20]. Although these studies have shown improvements in antibiotic prescribing, possible problems related to implementation and use of the system by physicians were not taken into account. Therefore, it is not clear whether these results can be repeated in a “real clinical setting” that we used. Another strength of this study is that a report is given on how stakeholders were involved before implementation to justify intervention design. Very few studies on CDSS report pre-deployment stakeholder analysis [8]. Other developed CDSS for empirical antibiotic therapy were tested in a limited number of departments [13, 17, 18], or only in hospitalized patients with bloodstream infection [16, 36] or pneumonia [18, 37]. Our CDSS was implemented in a tertiary hospital with a wide variation of departments, which has the risk of less focus on departments or diseases in which antibiotics are used most frequently. However, by targeting a broader population of physicians more use can be expected. In addition, physicians who prescribe less antibiotics benefit more from being assisted with antibiotic choices using this system, because of less experience in prescribing this medicines. For this reason we feel it is important to also include these prescribers.

A recently published study describes the development and implementation of a similar CDSS [35]. However, this CDSS is developed for primary care and is not linked to a hospital information system. This system is a less advanced system, which is not able to automatically extract data which makes the recommendations less individualized and accurate. In addition, it is not clear how often the system could have been used and what the real uptake of recommendations is, because details of antibiotic prescriptions were not collected (35).

## Conclusions

In conclusion, we developed a CDSS for empirical antibiotic therapy for adult hospitalized patients and gave a description of its development, validation and implementation. We have shown the usefulness of a systematic framework for reporting CDSS interventions. In addition, our data indicate that CDSS recommendations are not always adhered to and incorrect use of the system plays an important role in this.

## Supplementary Information

**Additional file 1.** The resistance viewer in the CDSS for empirical antibiotic therapy.

## Data Availability

Data will be available from the corresponding author H. Akhloufi (h.akhloufi@erasmusmc.nl) on reasonable request.
