# Supplementary material for: The development and implementation of a guideline-based clinical decision support system to improve empirical antibiotic prescribing
Source: BMC Med Inform Decis Mak. 2022 May 10;22:127. doi: 10.1186/s12911-022-01860-3 (PMC9087957; doi:10.1186/s12911-022-01860-3)

**Additional file 1**

Figure S1. Complete flowchart of high and moderate risk community acquired pneumonia

Figure S2. The resistance viewer in the CDSS for empirical antibiotic therapy


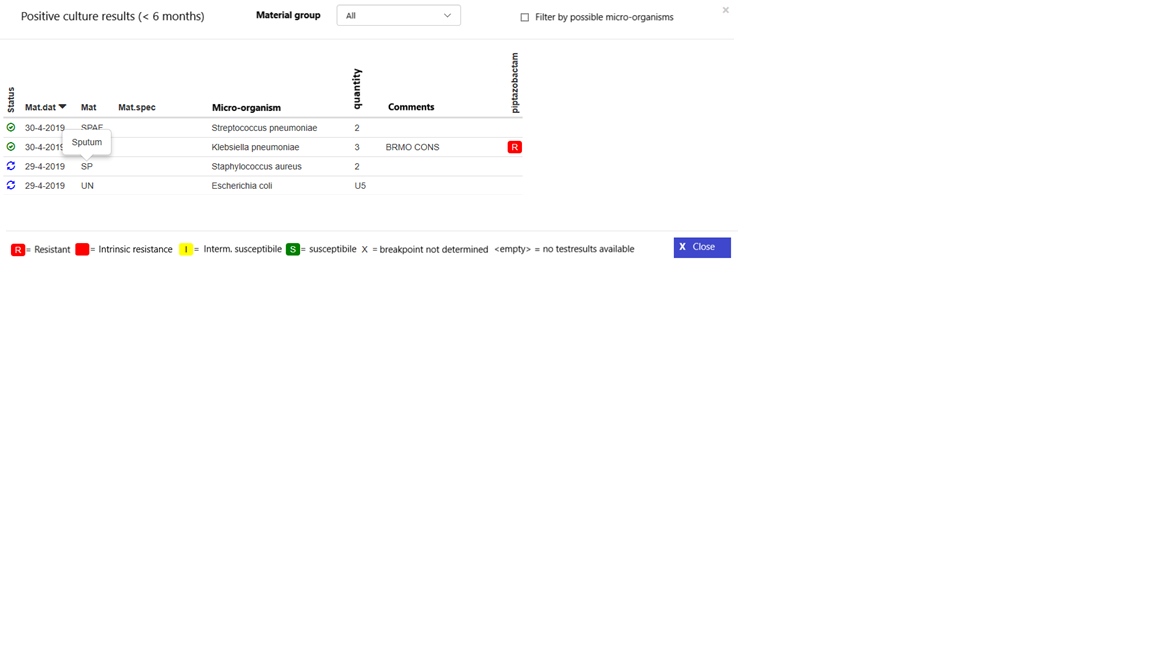

Supplement: Supplementary file 1 — Additional file 1. The resistance viewer in the CDSS for empirical antibiotic therapy. [file 12911_2022_1860_MOESM1_ESM.doc]
